# Evaluation of dendritic cells loaded with apoptotic cancer cells or expressing tumour mRNA as potential cancer vaccines against leukemia

**DOI:** 10.1186/1471-2407-5-20

**Published:** 2005-02-18

**Authors:** Silvija Jarnjak-Jankovic, Rolf D Pettersen, Stein Sæbøe-Larssen, Finn Wesenberg, Gustav Gaudernack

**Affiliations:** 1 Department of Pediatric Research, The National Hospital, Oslo, Norway; 2 Section for Immunotherapy, The Norwegian Radium Hospital, University of Oslo, Norway; 3 Department of Pediatrics, The National Hospital, Oslo, Norway

## Abstract

**Background:**

Leukemia is a clonal disorder characterized by uncontrolled proliferation of haematopoietic cells, and represents the most common form of cancer in children. Advances in therapy for childhood leukemia have relied increasingly on the use of high-dose chemotherapy often combined with stem-cell transplantation. Despite a high success rate and intensification of therapy, children still suffer from relapse and progressive disease resistant to further therapy. Thus, novel forms of therapy are required.

**Methods:**

This study focuses on dendritic cell (DC) vaccination of childhood leukemia and evaluates the in vitro efficacy of different strategies for antigen loading of professional antigen-presenting cells. We have compared DCs either loaded with apoptotic leukemia cells or transfected with mRNA from the same leukemia cell line, Jurkat E6, for their capacity to induce specific CD4+ and CD8+ T-cell responses. Monocyte-derived DCs from healthy donors were loaded with tumor antigen, matured and co-cultured with autologous T cells. After one week, T-cell responses against antigen-loaded DCs were measured by enzyme-linked immunosorbent spot (ELISPOT) assay.

**Results:**

DCs loaded with apoptotic Jurkat E6 cells or transfected with Jurkat E6-cell mRNA were both able to elicit specific T-cell responses in vitro. IFNγ-secreting T cells were observed in both the CD4+ and CD8+ subsets.

**Conclusion:**

The results indicate that loading of DCs with apoptotic leukemia cells or transfection with tumour mRNA represent promising strategies for development of cancer vaccines for treatment of childhood leukemia.

## Background

Leukemia represents the most common form of cancer in children. There are two main types of childhood leukemia, acute lymphoblastic leukemia (ALL) and acute myeloid leukemia (AML). The success rate in treatment of childhood leukemia has improved continuously over the past decades [1], and today disease-free survival is 70%–80% for ALL and 40%–60% for AML [2-4]. In the Nordic countries the overall event-free survival in ALL has risen from 57% to75% [2]. However, in children with high-risk ALL, the progress has only been modest. The relapse rate has decreased in parallel with the improving results, but the prognosis after relapse has not improved. Only 25%–30% of children who relapse will reach and remain in a second remission. Children with AML have a worse prognosis than those with ALL. Event-free survival for AML is below 55%, whereas the cure rate for children with ALL is near 80%. The complete remission rate differs also, with 5%–10% induction failures due to refractory disease and toxicity in AML, compared to 1%–2% in ALL [2].

Immunotherapy based on vaccination with dendritic cells (DCs) has emerged as an attractive new form of therapy for cancer in general, and DC-based vaccines have already shown promise in follicular non-Hodgkin's lymphoma, and in other hematological malignancies [5-7]. DCs are antigen-presenting cells (APCs) specialized to induce T-cell responses against cells exposing foreign peptides, including tumour-related antigens, in context of MHC molecules [8,9]. DCs reside in tissues in an immature form, where they capture antigens from the environment. After antigen capture, and in response to inflammatory stimuli, DCs mature and migrate to lymph nodes to initiate immunity [9]. Maturation of DCs is associated with up regulation of the co-stimulatory molecules CD80 and CD86, increased expression of HLA molecules, enhancement of their APC function, and expression of CCR7 chemokine receptors that promote migration to the T-cell area in lymph nodes [10]. In several animal studies, it has been shown that immunization with cancer-antigen loaded DCs efficiently primes both CD4+ and CD8+ T-cells, resulting in protective immunity against tumours [11-16].

Vaccination with tumor antigen-loaded DCs has been shown to induce both Th and CTL responses, and tumor regression in some patients [16,17]. An important issue in optimizing DC vaccines is the choice of tumour antigen for loading of DCs. Several clinical trials in patients with melanoma have demonstrated that vaccination against a single antigen can induce tumour specific CTLs [18]. However, for many tumours no specific cancer antigens are known. For such patients, autologous tumor cells or tumor cell lines containing a repertoire of antigens overlapping with the repertoire in the patient's tumor, represents an alternative source of antigens. Effective cross-priming with antigens from tumour cells has been demonstrated with apoptotic cancer cells [19-21]. Transfer of whole tumor mRNA into DCs represents an alternative way of loading DCs. The transfected mRNA can be expressed for a relatively long period of time [22-24] and give rise to specific T-cell responses in vitro and following vaccination of patients [25]. So far, no studies on the relative efficacy of these two antigen loading methods have been performed. The aim of the present study was to compare DCs either loaded with apoptotic Jurkat E6 cells or transfected with mRNA isolated from Jurkat E6 cells, for their ability to generate T-cell responses against antigens derived from the human T-cell line. The Jurkat leukaemic T-cell line is a reference cell line [26], and was chosen as a source of antigen in the model experiments described here. We demonstrate that both strategies can be successfully employed to induce T helper and CTL responses against antigens derived from allogeneic leukemic T-cells.

## Methods

### Cytokines and chemicals

GM-CSF was purchased from Novartis (Basel, Switzerland), IL-4, TNFα, IL1β and IL-6 from CellGenix (Freiburg, Germany), and Prostaglandin E2 (PgE_2_), IL-7, IL-2 and IL-12 from R&D Systems (Minneapolis, USA). Staurosporin was obtained from (Sigma Aldrich, Saint Louise, Missouri).

### Preparation of DCs and T cells

PBMC from healthy donors (obtained from Buskerud Hospital, Drammen, Norway) were obtained by density gradient centrifugation (Lymphoprep, Nycomed, Norway). Monocyte-derived DCs were generated under serum-free conditions from the adherent fraction of PBMCs cultured in six-well plates at a density of 4 × 10^6 ^cells/ml for 1.5 h at 37°C in 3 ml CellGro DC medium (CellGenix, Freiburg, Germany). Non-adherent cells were collected and frozen for later use as responder cells. Adherent cells were cultured in 3 ml CellGro DC medium, supplemented with 800 U/ml GM-CSF and 10 ng/ml IL-4 every second day, until day 5 when maturation of DCs was induced by addition of maturation cocktail (10 ng/ml TNF-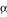, 10 ng/ml IL-1
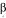
1000 U/ml IL-6 and 1 μg/ml PgE_2_) for 24 h. Characterization of DC phenotype was done by staining 0,5 × 10^6 ^cells with fluorochrome-labelled antibodies against the Lin1 panel (CD3, CD14, CD19, CD16, CD20, CD56), HLA-DR, CD1a, CD80, CD83, and CD86 (Becton Dickinson, San Jose, CA), and analyzing by FACSCalibur flow cytometry (Becton Dickinson). The mAb isotypes used were IgG1 FITC, IgG2a PE, IgG1 APC (Becton Dickinson, San Jose, CA).

### Assessments of apoptosis and phagocytosis of apoptotic cells

Jurkat E6 cells obtained from American Type Culture Collection (ATCC) were exposed to 1 μM staurosporin for 3 h to induce apoptosis. Apoptotic cell death was assessed using Annexin V-FLUOS as described by the manufacturer (Boehringer Manheim, Manheim, Germany). For assessments of phagocytosis, Jurkat E6 cells were stained with the green fluorescent dye PKH-67 (Sigma Aldrich) as described in the kit manual, exposed to 1 μM staurosporin for 3 h and incubated with immature DCs at a ratio of 3:1. After 6 h, immature DCs were labelled with a red fluorescent antibody (mAb CD1a-PE). Phagocytosis of apoptotic cells was measured quantitatively by flow cytometry. Similarly, phagocytosis was visualized by confocal laser microscopy (Leica TCS SP, equipped with HeNe and Ar lasers) using apoptotic Jurkat E6 cells pre-stained with PKH-26 red fluorescent dye (Sigma Aldrich) and DCs stained with the green fluorescent dye PKH-67.

### Preparation of Jurkat E6-cell mRNA and transfection of DCs

Jurkat E6 cells were used as a source of tumor material. Total RNA was isolated from 20–25 × 10^6 ^cells using Trizol Reagent as described by the manufacturer (Invitrogen, Basel, Switzerland). Poly (A)^+ ^mRNA was isolated from total RNA using the GenoPrep Direct mRNA kit (GenoVision, Oslo, Norway). Purified mRNA was used fresh or stored at -80°C until use. Transfection of DCs with mRNA was performed as described previously [22], with minor modifications. Briefly, immature DCs were washed once, resuspended in RPMI-1640 (BIO-Whittaker, Walkersville, MD) and placed on ice. 400 μl (approx. 2 × 10^6 ^cells) were mixed with mRNA, transferred to a 4-mm-gap cuvette and pulsed with a BTX ECM-830 square-wave electroporator (Genetronics Inc., San Diego, CA) using instrument settings 500 V and 1 ms. Transfected cells were incubated on ice for 30 s followed by addition of 2.0 ml cold CellGro DC medium supplemented with 10 ng/ml IL-4, 800 U/ml GM-CSF and maturation cocktail (see above), and transferred to standard culturing conditions. Transfection with EGFP-pCIpA_102 _mRNA (10 μg/400 μl) encoding the green fluorescence protein [22] was used to verify transfection efficiency.

### Isolation of T-cell subsets CD4 and CD8

The Negative Isolation Kit (Dynal, Biotech) was used for isolation of CD4 and CD8 T cells according to the manufacturer's protocol. Isolation was performed on day 7 after in vitro priming, before setting up the ELISPOT assay.

### Induction of primary T-cell responses

Mature DCs (0.3 × 10^6^) expressing Jurkat E6-cell mRNA or loaded with apoptotic Jurkat E6 cells, were co-cultured with 3 × 10^6 ^autologous non-adherent PBMC for 7 days in 1.0 ml CellGro DC medium without serum, before setting up the ELISPOT assay. The cultures were tested for INF-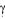 production in an ELISPOT assay [27] following restimulation for 24 h with thawed antigen-loaded DC using 0.5 × 10^5^, 1.0 × 10^5^, 2.0 × 10^5 ^and 4.0 × 10^5 ^responding cells and 0.5 × 10^4 ^DCs per well. Mock transfected DCs were used as control. The assay was done in duplicate. Spots were counted manually, and the frequency of reactive T cells was calculated according to the formula: (spots with transfected DC - spots with non transfected DC)/number of T cells added.

## Results

### Generation of immature DCs and phagocytosis of apoptotic Jurkat E6 cells

**Figure 1:**
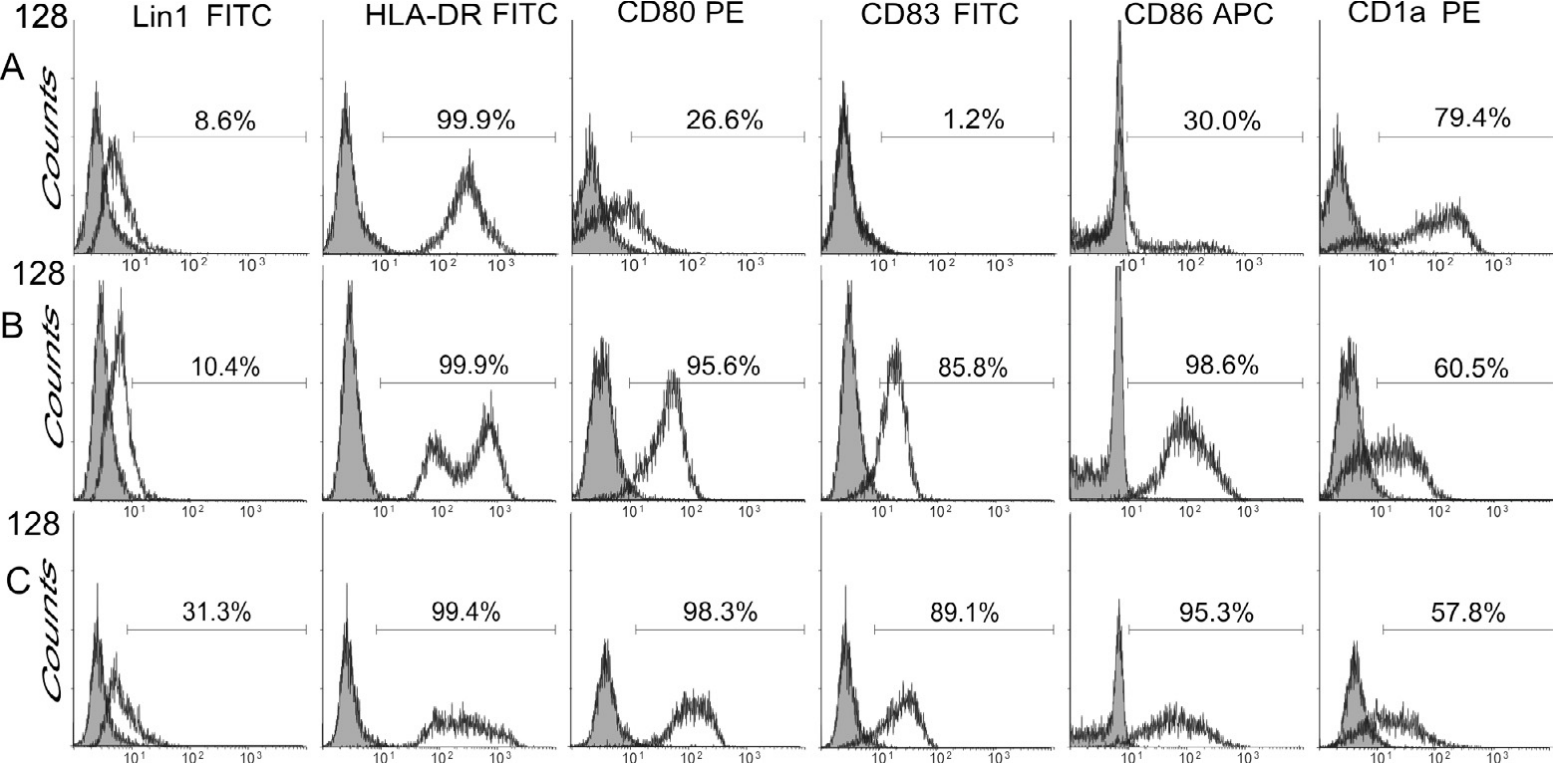
Phenotype of generated DCs. Expression of antigens were determined by flow cytometry (a) Before loading with tumor antigens (b) After loading with apoptotic Jurkat E6-cells and following maturation with TNFα, IL1β, IL6 and PgE2 for 24 h and (c) After transfection with Jurkat E6-cell mRNA and maturation for 24 h. The histograms show staining with the appropriate mAb.

Based on previous observations that immature DCs efficiently capture antigens from the environment, we first investigated the ability of immature DCs to similarly phagocytose apoptotic Jurkat E6 cells. Immature DCs were prepared from PBMC in the presence of IL-4 and GM-CSF. To confirm generation of immature DCs, cells were examined by flow cytometry for expression of lineage and differentiation specific markers. The Lin 1 cocktail contains antibodies to CD3, CD14, CD16, CD19, CD20, and CD56 and differentiates DCs from other leukocytes by their lack of staining with Lin1. In contrast, CD1a and HLA-DR are expressed on immature DCs, and maturation is revealed by increased expression of CD83, and the costimulatory molecules CD80 and CD86. As shown in Fig. 1a the generated cells displayed the characteristic phenotype of immature DCs with high expression of CD1a and HLA-DR, and low or no expression of CD80, CD83 and CD86.

**Figure 2:**
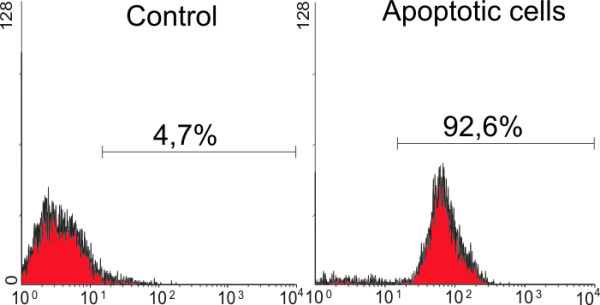
Staurosporin-induced apoptosis of Jurkat E6-cells. T cells were cultured for 3 h with 1 μM staurosporin and examined by Annexin V-FLUOS staining and flow cytometry.

For induction of apoptosis, Jurkat E6 cells were treated with staurosporin. In an independent series of experiments, optimal early apoptosis was observed after 3 h (data not shown) and early apoptotic cells were accordingly used in all experiments. To verify apoptosis after 3 h exposure to staurosporin, cells were stained with annexin V-FLUOS and examined by flow cytometry. The assessments showed that more than 90% of the cells were annexin-V positive (Fig. 2).

**Figure 3:**
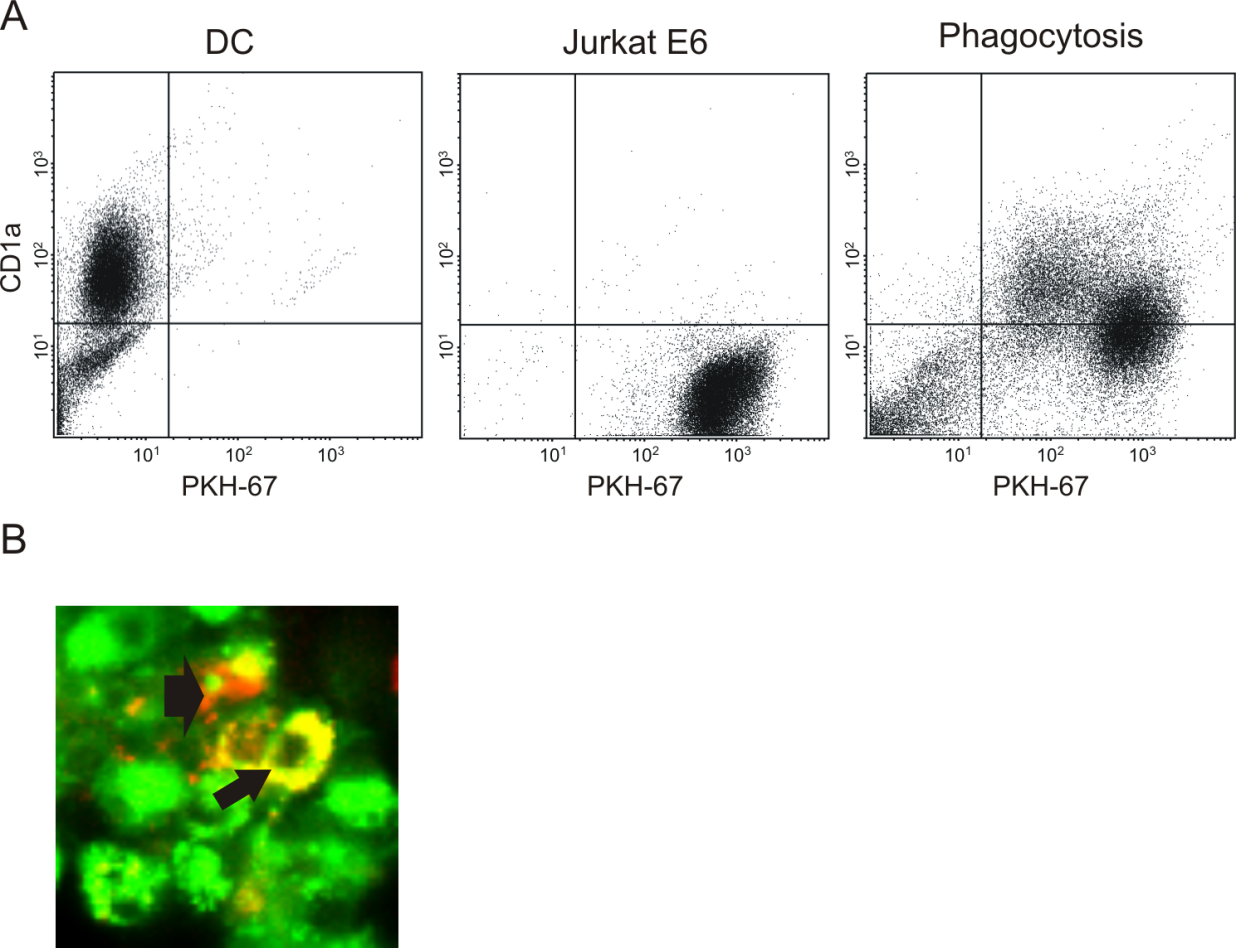
Phagocytosis of apoptotic Jurkat E6 cells by immature DCs. At day 5 of culture, DCs were co-cultivated with apoptotic Jurkat E6 cells, previously labelled with green fluorescent dye. (a) Flow cytometry of immature DCs stained with anti CD1a-PE and apoptotic Jurkat E6 cells stained with PKH-67 green. (b) Confocal microscopy analysis shows the presence of intracellular apoptotic cells labelled with PKH-26 red within DCs labelled with PKH-67 green (yellow cell, arrow) or in the process of being phagocytized (yellow and red cell, arrow).

To study uptake of apoptotic leukemia cells by immature DCs, Jurkat E6 cells were stained with the red fluorescent dye PKH-26 before induction of apoptosis. Immature DCs were stained with the green fluorescent dye PKH-67. Apoptotic Jurkat E6 cells were then co-incubated with immature DCs for various periods of time to determine the optimal conditions for internalization of apoptotic Jurkat E6 cells. We observed that immature DCs ingested apoptotic Jurkat E6 cells within 6 h of co-incubation. Phagocytosed apoptotic Jurkat E6 cells (red stained) were observed inside or in the process of being phagocytozed by immature DCs (green stained) by confocal microscopy (Fig. 3b).

Flow cytometry was used to further determine the efficiency of DC loading with apoptotic leukemic cells. In these experiments, apoptotic Jurkat E6 cells had been pre-stained with the green fluorescent dye PKH-67 and DCs were identified by staining with PE-conjugated anti-CD1a. Highly efficient uptake of apoptotic Jurkat E6 cells was confirmed, since virtually all CD1a positive cells showed green PKH-67 staining (Fig. 3a).

Following antigen loading, DCs were matured in the presence of pro-inflammatory cytokines for 24 h. Assessments by flow cytometry confirmed that this treatment led to up-regulation of CD83, and the co-stimulatory molecules CD80 and CD86, in compliance with a mature DC phenotype (Fig. 1b).

### Transfection of immature DC with mRNA from Jurkat E6 cells

**Figure 4:**
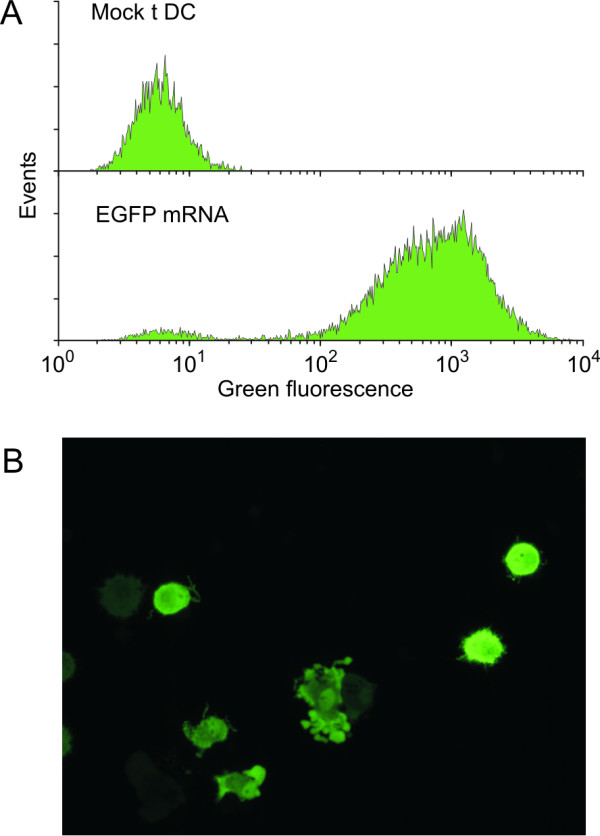
mRNA transfectation of DCs. (a) Flow cytometric analysis of DCs after transfection with EGFP/pCIpA_102 _mRNA (10 μg/400 μl) by square-wave electroporation and maturation for 24 h in medium with maturation cocktail. Control cells were mock electroporated without mRNA. (b) Fluorescence microscopy of DCs 24 h after transfection with EGFP/pCIpA_102 _mRNA.

Transfection of cells with tumor-derived mRNA is an alternative method for loading of DCs with tumor antigens. mRNA from the Jurkat E6 cells was isolated and electroporated into DCs according to previously optimized methods [22]. Following this protocol, optimal conditions for electroporation were 500 volt and 1 ms when using a 4-mm-gap cuvette. These conditions produced both efficient transfections (142 × background fluorescence; Fig. 4) and a survival rate indistinguishable from untransfected cells (data not shown). The transfected DCs were matured as described above and induction of the characteristic phenotype was confirmed by flow cytometry (Fig. 1c).

### Analysis of T-cell responses

**Figure 5:**
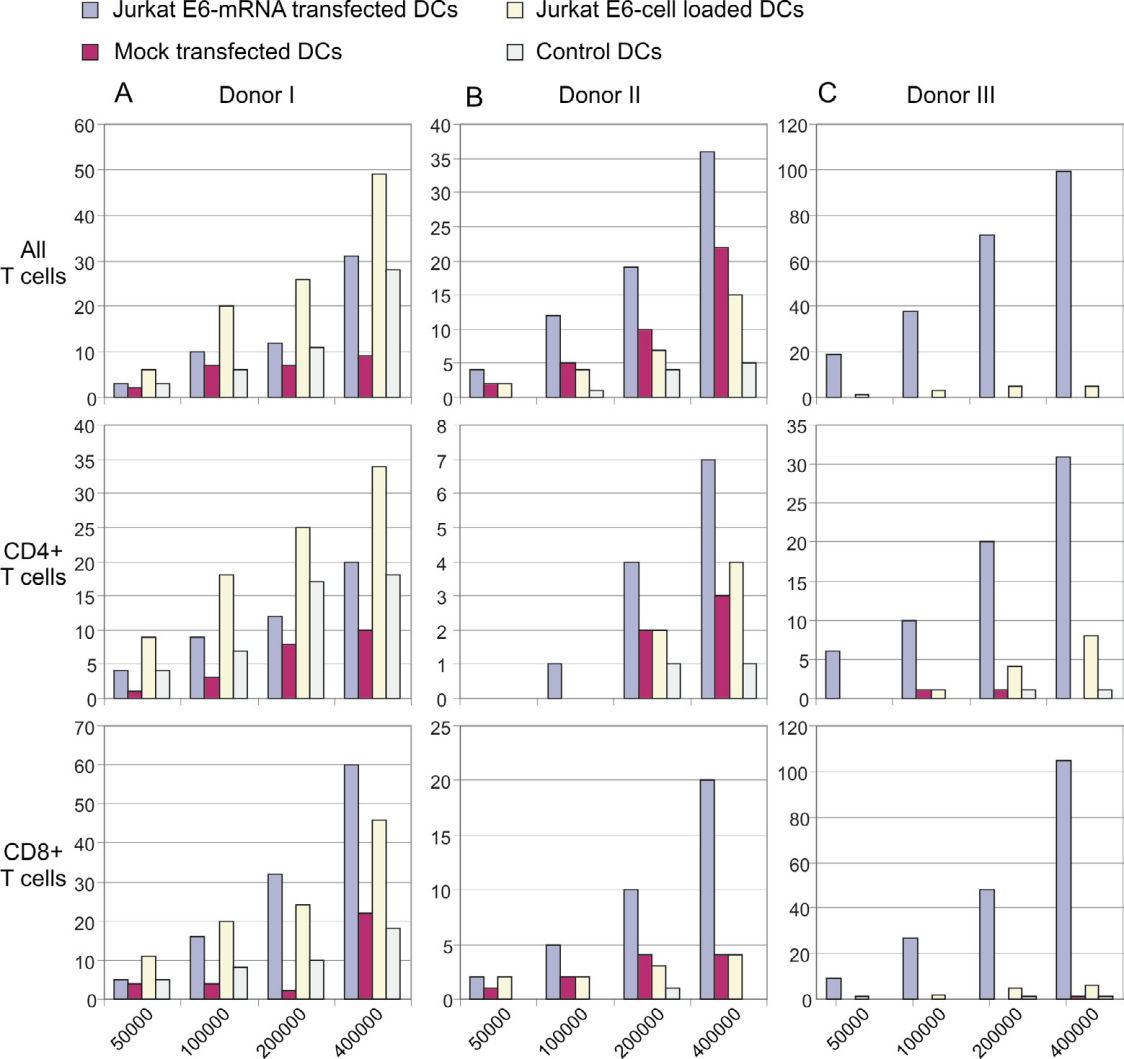
ELISPOT analysis of T-cell activation. T cells were stimulated with DCs loaded with tumour antigen (mRNA or apoptotic cells) or control DCs as indicated. (a) Mean number of IFN-γ positive spots obtained with the indicated numbers of un-fractionated T cells, and CD4+ and CD8+ T-cell subsets from individual donors. (b) Frequency of reactive T cells. The data represent mean values of all donors calculated by the formula: [(spots loaded DC - spots control DC)/10^5 ^T cells].

ELISPOT assay of INF-γ producing cells is the method of choice for assessments of T-cell responses against cancer vaccines representing a heterogeneous mixture of antigens. This assay measures in a quantitative way the number of reactive T cells in pre and post-vaccination samples and thus directly relates the effect of vaccination to in-vivo expansion of reactive T cells. Autologous T cells were stimulated with tumour-mRNA transfected DCs and with DCs loaded with apoptotic Jurkat E6 cells. Fig. 5 shows the results from experiments with cells derived from three different donors. In all experiments a specific T-cell response against antigen-loaded DCs as compared to control DCs (mock transfected/non-loaded) could be demonstrated. No clear-cut difference between the two modes of antigen-loading was observed. In all experiments with un-fractionated T cells, we also observed T-cell reactivity against control DCs. This background completely obscured the specific response if in vitro priming was performed in the presence of exogenously added recombinant human IL-2 (results not shown). Antigen loading of DC by mRNA transfection and phagocytosis will introduce the Jurkat antigens into two different antigen processing pathways, cytosolic expression and processing for mRNA-encoded antigens and endosomal processing for phagocytosed apoptotic cells. As a result, one might expect that mRNA loading would preferentially result in activation of specific CD8+ CTLs, while loading with apoptotic cells would mainly result in activation of specific CD4+ Th1 cells. To investigate if this was the case, we separated the responding T-cell population into a CD4+ containing fraction and a CD8+ containing fraction using negative selection with Dynabeads coated with CD8 and CD4 antibodies respectively. The results shown in Fig. 5 demonstrate that a specific Th1 response was obtained in all donors and that both methods of loading resulted in a Th1 response. The frequency of specific Th1 cells varied between donors, with a trend indicating that mRNA loading in general is more efficient than apoptotic cells in priming of a Th1 response. The results depicted in Fig. 5 clearly show that relatively high frequencies of specific CTLs can be generated in all donors and that both methods of antigen-loading result in CTL priming. In donor 2 and 3, mRNA loading was clearly superior to apoptotic cells, indicating that expression of mRNA-encoded antigens more efficiently entered the proteasomal pathway for processing of HLA class I restricted antigens.

## Discussion

Immunotherapy for childhood leukemia has the potential to contribute to long-term control or cure of the disease. Until now immunotherapeutic approaches for leukemia have been limited to trials of cytokine therapy [3]. Further development of biologically based treatments may prove to be effective in therapy of patients suffering from this disease. Several forms of DC-mediated immunotherapy are currently being investigated using a wide variety of vaccination protocols summarized in [28]. Two very important issues are the choice of antigen and the method of antigen loading. In the present study we have chosen to use the complex antigen mixture represented by whole tumor cells. Reports comparing the ability of apoptotic and necrotic cells to induce DC maturation [29] found that incubation of DCs with necrotic, but not apoptotic, tumor cell lines induce maturation. However, other reports concluded that incubation with apoptotic cells is sufficient to induce DC maturation [30-34]. In our study we have used apoptotic cells, and the requirement for DC maturation signals was provided by a standardized maturation cocktail. We accordingly analyzed human monocyte-derived DCs for their ability to: (a) take up apoptotic leukemia cells and express transfected mRNA, (b) express a mature phenotype following tumour-antigen capture and culture in maturation cocktail and (c) prime un-fractionated T cells as well as the CD4+ and CD8+ T-cell subsets. Due to the complexity of the antigens represented by the allogeneic tumor cells, the aim of these model experiments was not to use this allogeneic system to prove that we could elicit tumour specific T-cell responses in this way, but to provide data to demonstrate efficient antigen transfer and compare the relative efficacy of DCs loaded by the two different procedures, in eliciting complex T-cell responses. Our results demonstrate that immature DCs can efficiently take up apoptotic Jurkat E6 cells, and that phagocytosis was mainly confined to the CD1a+ subset of immature DC. Furthermore, support for expression of transfected mRNA derived from the allogeneic leukemia cell line is indirectly provided by its ability to prime T-cell responses specific for transfected cells. We also show that the two different methods of antigen-loading did not result in any apparent differences in the phenotype of the mature DCs. In terms of immune responses both methods of antigen loading produced DCs capable of inducing INF-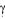 secreting T cells. However, it appeared that DCs loaded with tumour-mRNA in general were most potent in inducing T-cell responses.

We observed that the frequencies of induced INF-γ producing T cells depended on the individual donor, the method of antigen-loading and the subset of T cells studied. Such variations are not surprising, since the model system used employs allogeneic cells and no effort was done to HLA match the blood donors with the Jurkat cell line in these experiments. Since the experimental system is based on the use of an allogeneic cell line, we expect multiple antigens, encoded by a broad array of polymorphisms, including other HLA alleles to be involved. We have therefore taken advantage of the genetic differences between responding cells and the leukaemia cell line by using the combined repertoires of membrane expressed and cross presented allo-antigens as a sensitive readout for immunological response in our experiments.

We expected that the two different procedures would provide some differences in loading of HLA molecules with tumour-derived antigens and subsequently in the responding T-cell subsets. According to the current dogma, processed peptides from phagocytosed apoptotic cells would be directed to HLA class I molecules by a process known as cross-presentation and to HLA class II molecules by the classical pathway. Cross-priming of CTL with antitumor activity has been demonstrated with DCs loaded with apoptotic tumour cells [19,21,35]. Specifically, Schnurr et al. demonstrated the antigens from apoptotic pancreatic carcinoma cell lines, either in form of whole cells or as released particles, were potent in inducing CTL-cell priming and activation by DC. In addition, Hoffman et al. reported stronger CTL responses with apoptotic tumour cells in a squamous cell carcinoma model. The enhanced CTL activation by antigens from apoptotic cells may be attributed to several mechanisms. After ingestion, most particulated antigens requiring phagocytosis are digested into peptides associating with HLA class-II molecules in the endocytic compartments and are presented to T-helper cells [36]. Conversely, scavenger receptor-mediated phagocytosis of apoptotic tumour cells allows antigens to gain access to HLA class-I compartments, resulting in cross-presentation of the antigens to CTL [20]. In addition, enhanced CTL responses to tumours might be mediated by heat shock proteins expressed by stress induced apoptotic tumour cells [37]. On the basis of this theoretical background and reported observations [21,31] we believe that antigen preparations from apoptotic tumour cells can also represent an alternative in DC-based tumour vaccines. On the other hand, tumour mRNA expressed in DCs would be processed for presentation by HLA class I molecules. In accordance with this, DCs loaded with apoptotic leukaemia cells stimulated both CD4+ and CD8 positive T cells and mRNA loaded DCs were superior in inducing CD8+ T-cell responses [38,39]. Interestingly, mRNA-loaded DCs were also able to induce specific CD4+ T-cell responses in all donors tested, suggesting some leakage of endogenously produced proteins into the lysosomal antigen-processing compartments. Similar results have recently been published by Su et al., who demonstrated a significant Th response against the defined tumour antigen hTERT following in vitro stimulation of un-fractionated T cells with hTERT mRNA transfected DC. The Th response could be further augmented by targeting the antigen to the lysosomal compartment using mRNA encoding a chimeric hTERT/lysosome-associated membrane protein (LAMP-1) protein.

The aim of the present study was to determine if loading of DCs with antigens derived from a tumour cell line, either as apoptotic cells or as mRNA would provide a basis for an efficient vaccine, using ELISPOT as a read-out of immune responses. It has been shown that DCs transfected with antigens encoded in tumor mRNA is capable of inducing potent T-cell responses against tumour-specific epitopes [40]. While protein antigens from tumour lysate are rapidly proteolysed following endocytosis by antigen-presenting cells, model experiments using mRNA encoding a fluorescent protein, EGFP, has shown that protein is still being produced 24 hrs after transfection of DCs, with peak expression after 48 hrs [22]. Thus, tumor mRNA transfected DCs may not only represent a potent strategy for CTL priming but may also represent a general method for DC-based vaccines. In vaccine preparations using DCs, mRNA is thus preferable to a protein lysate. Similarly, immunization with DCs loaded with mRNA from leukaemia cells could represent a feasible approach in treatment of these cancers. It is now widely accepted that not only CTLs but also CD4 (+) T-helper cells are critical to the generation and maintenance of potent antitumor responses in vivo. In this context, our observation and that of others demonstrating that DCs loaded with mRNA also are equally capable of inducing Th responses strongly argue in favour of this type of vaccination. Our preclinical results further support that vaccination of leukemia patients with tumour-mRNA transfected autologous DCs should be clinically evaluated as therapeutic strategy.

## Conclusion

In our study we demonstrate that both DCs loaded with apoptotic Jurkat E6 cells or transfected with mRNA isolated from Jurkat E6 cells, can induce T-helper and CTL responses against antigens derived from allogeneic leukemic T-cells. We also show that the two different methods of antigen-loading did not result in any apparent differences in the phenotype of the mature DCs. In terms of immune responses both methods of antigen loading produced DCs capable of inducing INF-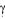 secreting T cells. However, it appeared that DCs loaded with tumour mRNA in general were most potent in inducing T-cell responses.

## Competing interests

The author(s) declare that they have no competing interests.

## Authors' contributions

SJJ performed preparation of DCs and T cells, assessments of apoptosis and phagocytosis of apoptotic cells, preparation of Jurkat E6-cell mRNA and transfection of DCs, isolation of T-cell subsets CD4 and CD8 and induction of primary T cell responses. SSL did the transfection of DCs with EGFP mRNA and fluorescence microscopy of DCs. RP, FW and GG planned the project.

## Pre-publication history

The pre-publication history for this paper can be accessed here:
